# Immunocrit, serum amino acid concentrations and growth performance in light and heavy piglets depending on sow’s farrowing system

**DOI:** 10.1186/s40813-019-0121-1

**Published:** 2019-06-14

**Authors:** Sandra Schnier, Lea Middendorf, Heiko Janssen, Carla Brüning, Karl Rohn, Christian Visscher

**Affiliations:** 1 Institute for Animal Nutrition, University of Veterinary Medicine Hannover, Foundation, Bischofsholer Damm 15, D-30173 Hannover, Germany; 2 Chamber of Agriculture of Lower Saxony, Mars-la-Tour-Str. 6, D-26121 Oldenburg, Germany; 3 Institute for Biometry, Epidemiology and Information Processing, University of Veterinary Medicine Hannover, Foundation, Bünteweg 2, D-30559 Hannover, Germany

**Keywords:** Farrowing system, Piglets, Body weight, Serum, Immunocrit, Amino acid, Performance

## Abstract

**Background:**

The conventional farrowing crate is criticised due to the limited mobility of sows during farrowing and lactation. The present study aims to investigate the effects of three different farrowing systems on the performance of suckling neonates on the basis of immunocrit (IC; a quantification of immunoglobulins), serum amino acid (AA) concentrations and growth performance.

**Methods:**

From a total of 149 sows placed in three housing systems (farrowing crate – FC, loose housing – LH, group housing – GH), 18 sows and their respective litters, formed the basis for a two-factorial study design (farrowing system and body weight (BW) of neonates). Therefore, also blood samples of two light (1.0–1.4 kg) and two heavy (≥ 1.4 kg) piglets were taken within 48 h post natum (p.n.) and on the day of weaning (day 26) to determine the immunocrit (IC; a quantification of immunoglobulins) and levels of serum AAs.

**Results:**

The IC (FC: 0.148^a^, LH: 0.153^a^, GH: 0.117^b^) as well as serum levels of arginine, leucine, lysine, proline and threonine within 48 h p.n. were significantly lower in GH. Additionally, in general, these piglets showed (except for the first week of life) the lowest average daily weight gain. On the day of weaning, piglets in GH had the lowest levels of arginine (in mg/dL; FC: 3.68^a^, LH: 3.40^ab^, GH: 2.94^b^) and threonine (in mg/dL; FC: 3.59^a^, LH: 3.02^ab^, GH: 2.49^b^). The concentrations of leucine, lysine, proline and valine at this time were significantly lower in LH.

**Conclusion:**

The observed significant lower IC indicates a lower Ig intake of piglets in the tested GH. No significant differences regarding the IC and AA levels within 48 h p.n. of the piglets in FC and LH could be seen. In principle, differences at weaning in AA levels were rather small, although the body weight of GH piglets at weaning was lower. Therefore, further research needs to clarify whether there are medium-term effects on health and performance.

**Electronic supplementary material:**

The online version of this article (10.1186/s40813-019-0121-1) contains supplementary material, which is available to authorized users.

## Background

Piglet losses occur mainly in the first three days after birth [1, 2]. A major cause of neonatal mortality is crushing by the sow [3], which is primarily predisposed by low BWs and low colostrum intakes of piglets [4, 5]. Directly after birth, glycogen from liver and muscle stores provides energy to the piglets [6, 7]. The low glycogen reserves are only sufficient for normal activity of the newborn piglets for the first 16 h after birth if no colostrum is ingested [2]. About 10% of the glycogen is in the liver, the remaining 90% being in the muscle tissues of the piglets [8]. Selecting sows for increased litter sizes leads to more piglets with low BWs and decreasing body energy stores at birth [9]. Furthermore, low-BW piglets are less competitive at the udder and could obtain less colostrum from the teats compared to their heavier littermates [10]. More precisely, the higher the birth weight, the better the performance and survival rate of piglets [9, 11]. Colostrum is the first milk from the mammary gland and the sole external source of nutrients, which should be taken in by newborn piglets shortly after birth [2, 10, 12–14]. It is important for growth and thermoregulation [15, 16]. A minimum intake of 250 g per piglet is recommended [17]. Colostrum is needed to support the passive transfer of immunity [10]. Newborn piglets are reliant on the colostrum immunoglobulin which supports the underdeveloped immune system [18, 19]. Thus, the survival rate of piglets is positively correlated with the concentrations of immunoglobulin G in plasma [20]. It is reported that colostrum also affects intestinal development [21, 22]. Furthermore, colostrum is rich in AAs, which are also important, among other things, as ‘metabolic fuel’ for the gastrointestinal tract [23, 24]. Leucine, for example, could improve intestinal development [25] and has a high relevance for muscle protein synthesis in neonates [26, 27]. Arginine influences maximal growth of piglets [28]. Nutritionally essential AAs in pigs are proline (young pigs), arginine, histidine, isoleucine, leucine, methionine, phenylalanine, threonine, tryptophan and valine [29]. The essential AAs with the highest content in colostrum are proline and leucine [30]. In addition to body reserves, BW and the colostrum supply of piglets, another factor influencing the survival of neonates is the housing system of sows [31]. The use of farrowing crates for sows during farrowing and lactation is increasingly being discussed in Europe. Relatively little is known about how alternative housing systems could influence the piglets’ supply of colostrum and AAs in serum. The core question regarding animal husbandry is focused on aspects of animal protection and animal welfare. Loose-housed sows are more active [32], whereas free-farrowing systems lead to a higher risk of piglets being crushed by sows, this being unacceptable from an animal welfare point of view [33]. Newborns which have only absorbed a small amount of colostrum, are often too weak and do not notice when the sow changes her position [2]. Therefore, alternative housing systems have to be developed and tested especially with regard to the adequate supply of newborn piglets with colostrum. The objective of the current experiment was to study the effects of three different farrowing systems (farrowing crate, loose housing, group housing) of lactating sows on the IC, serum AA concentrations and growth parameters in light and heavy piglets.

## Methods

### Animals, housing, management and feeding

**Fig. 1:**
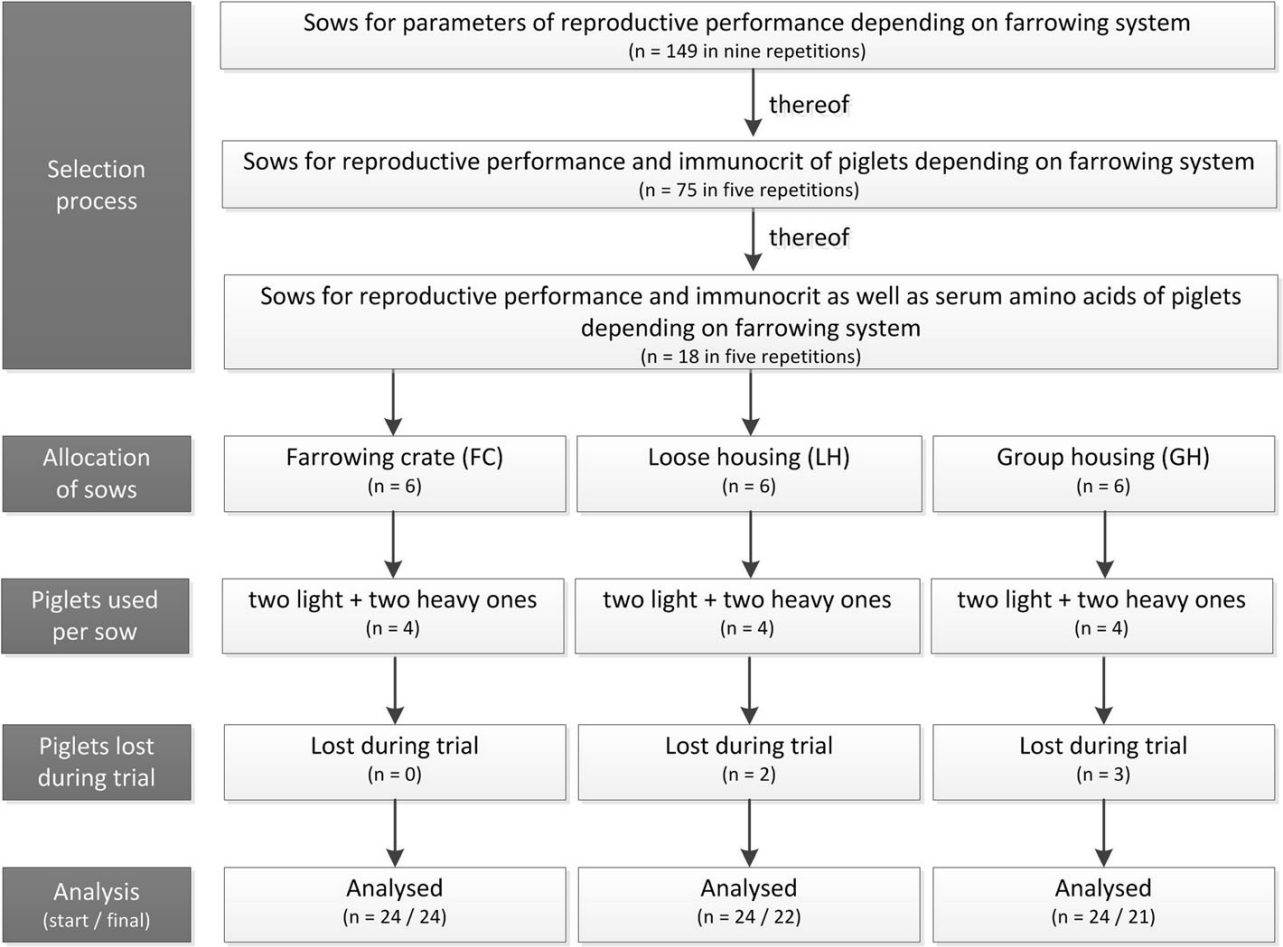
Trial flow diagram. The diagram indicates the selection process and number of animal losses

The experiments were performed in accordance with the German rules and regulations and approved by the Ethics Committee of Lower Saxony for the Care and Use of Laboratory Animals (LAVES: Niedersächsisches Landesamt für Verbraucherschutz und Lebensmittelsicherheit; reference: 33.19–42,502-05-16A020). The study took place on the pig farm of the Landwirtschaftskammer Niedersachsen (Chamber of Agriculture, Lower Saxony) in Wehnen, North Germany, in a moderate maritime climate zone. The facilities were rebuilt especially for this experiment. The experiments were carried out over a period of 12 months from September 2016 to August 2017*.* The farm kept about 80 reproductive sows of the db.Viktoria gene from the German Federal Hybrid Breeding Programme (BHZP GmbH, Dahlenburg-Ellringen, Germany) and batch farrowed at five-weekly intervals. The average suckling period was 26 days. The day of birth was defined as day 0 of age. In a total of nine trials (Fig. 1), 149 sows were randomly assigned to three different housing systems. In accordance with the permission to perform the animal experiment, blood samples were obtained from a total of 75 litters to test IC. For the results of amino acids analysis in serum, 18 sows were selected, which had nearly the same average parity and number of piglets born alive. A total of 30 light (1.0–1.4 kg) and 42 heavy (≥ 1.4 kg) piglets were included in the study. Four piglets were always used for sampling per litter. However, it was not always possible to take exactly two light and heavy piglets by definition, which meant that the number of piglets differed between weight classes.

**Fig.2:**
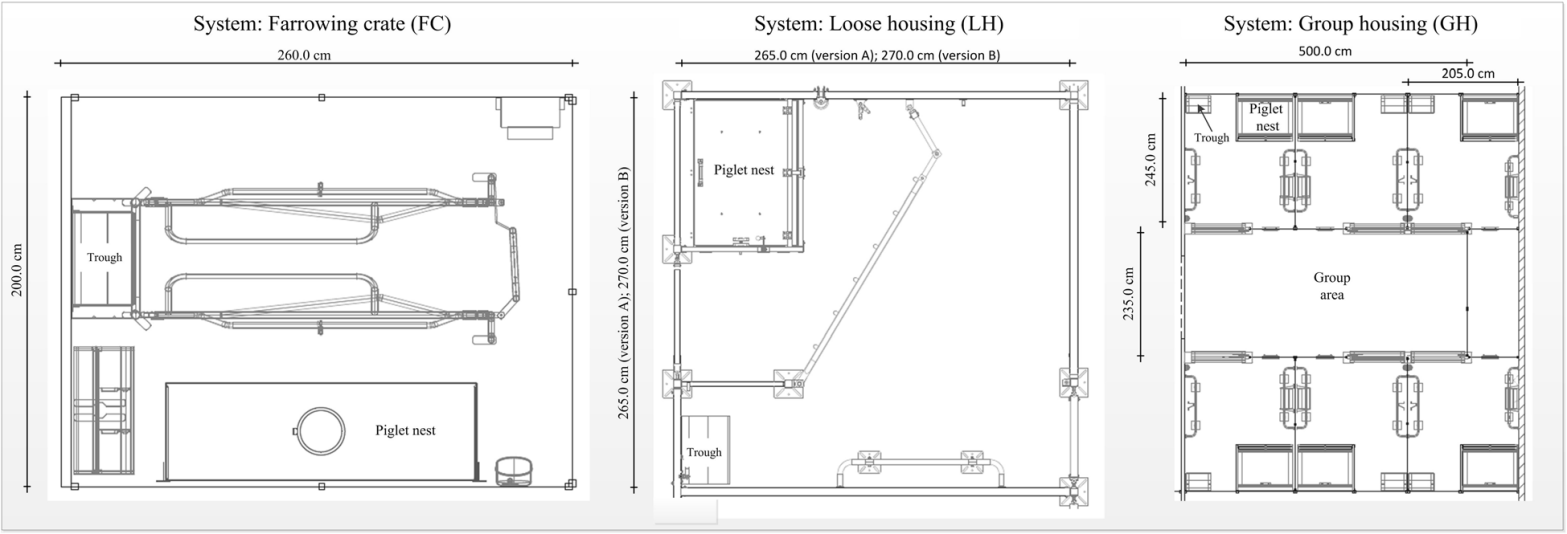
Schematic drawing of the three experimental farrowing systems. Length and width include the pen walls. (FC) farrowing crate, (LH) loose housing and (GH) group housing.^©^Big Dutchman

Two compartments were each equipped with the respective systems, which were used alternately (Big Dutchman International GmbH, Vechta, Germany; Fig. 2). Six days before expected parturition, all sows were moved to the farrowing rooms.

**Fig. 3:**
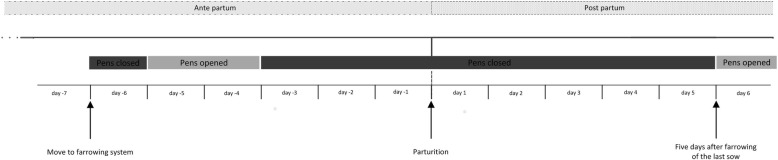
Time sequence between the day of stabling and five days post partum (p.p.) in group housing (GH)

The conventional single farrowing crates (FC) measured 260 cm in length and 200 cm in width. Eight FC pens were in one room. The FC was positioned in the centre of each pen. The floor of the pen in the area of the trough at the front end was equipped with non-perforated concrete flooring. There was fully slatted synthetic flooring made of plastic in the rest of the pen. The piglet nest (160 × 50 cm) was positioned at a side wall, was roofed and opened at three sides. It was equipped with a 150 W infrared heating lamp (different manufacturers) suspended above the piglet nest and with heating plates made of polymer concrete (Big Dutchman International GmbH, Vechta, Germany). This system was compared with a single loose housing (LH; six pens in one room), where the sows were individually confined and could move freely all the time. The sows could be fixed in exceptional cases by using a swing gate. This was attached in front of the piglet nest when it was not in use. The size of the pens measured 265 × 265 cm (version A) and 270 × 270 cm (version B), respectively. The LH pens had fully slatted plastic flooring. The piglet nest (100 × 80 cm) was positioned in the corner of the pen. This was a closed box with two entrances, which could be closed if necessary. The box was heated by an infrared heating element (maximal 270 W/hour; CE-REX IRX 300, Rexlan Europe, Sorø, Denmark). Rubber mat flooring was used in the piglet nest. Piglet protection bars were attached to two sidewalls. The other six sows were allocated to group housing (GH) with six individual pens (205 × 245 cm) and a group area (500 × 235 cm) between the pens. There was no possibility of fixing the sows. During the first 24 h after being moved to the farrowing unit and in the peripartal period (three days ante partum up to five days after farrowing of the last sow), the animals in this system were housed in the individual pens so that they had no access to the group area, but could move freely during farrowing and sampling (Fig. 3).

Each pen had fully slatted flooring. About half of the flooring was concrete. The remainder was covered with cast iron slats. The flooring in the group area was predominantly slatted concrete. The piglet nest was the same as used in LH. On all side walls where possible, piglet protection bars were installed. At the front end of the group area, there was a separated area only for piglets (110 × 235 cm).

Sows were fed a commercial lactation diet following a restricted feeding scheme (up to day 1 a.p. 3.60 kg as fed (FC, LH) and 5.00 kg as fed (GH). On the day of parturition (day 0), feed was reduced to 2 kg per day in all housing systems. Afterwards, the amount of offered feed was gradually increased (circa 0.50 kg as feed/day) until almost ad libitum was reached from day 15 p.p. onwards. Creep feed was offered to piglets from day 10 of lactation. Sows and piglets had free access to water. The litter size was standardised when necessary within a system between 24 h and 48 h p.n. On average, piglets were weaned at day 26. On the same day, the sows were moved to the gestation house. There, the sows were housed in groups of three to five sows with self-catching crates. The sows of the GH did not necessarily know each other from the gestation house.

### Data collection

Parity, total number of born, live-born and stillborn piglets as well as the number of piglets after litter equalisation, weaned piglets and piglet losses were recorded for each litter. The individual BW of the piglets was measured 24 h p.n., once a week and on the day of weaning with a scale (IP68 AGT, T.E.L.L.-Steuerungssysteme GmbH & Co. KG, Vreden: Germany). Daily weight gain of piglets and the litter growth were determined. The weights of the piglets which had died during the trials were taken into account when calculating the litter weight gain. Within 48 h p.n. and before weaning (day 26), blood samples (maximum: 4.0 mL) were obtained by *vena cava* puncture and jugular venous blood, respectively, from two light (> 1.00 kg - <  1.40 kg) and two heavy (≥ 1.4 kg) piglets taken from each litter. Blood samples were allowed to clot at room temperature, followed by centrifugation at 1500 g for 15 min. Serum was removed and frozen at − 20 °C until analysis.

### Analysis of serum

To provide an assessment of the amount of colostrum and the passive transfer of immunoglobulins (Ig) from the sow to the piglets, the immunocrit method was used [34]. The IC is a quantification of immunoglobulins. Therefore, 50 μL of a 40% (wt/vol) of ammonium sulphate was added to the serum to precipitate immunoglobulins. The samples were centrifuged for 10 min (11,000 g) in a microhaematocrit capillary (disposable microhaematocrit capillary tubes 75 mm/75 μL, Hirschmann Laborgeräte GmbH & Co. KG, Eberstadt, Germany). The IC was calculated as the ratio of the precipitate to the total length of the column.

To determine the AA concentrations in serum, 1000 μL serum were mixed with 250 μL sulfosalicylic acid solution. After the samples had been stored for 30 min at 4 °C, they were centrifuged (10 min, 13,000 g). The supernatant was then transferred to another reaction vessel, diluted with the same amount of dilution buffer and mixed thoroughly. Then, circa 350 μL of the samples were filtered. An AA analyser was used to determine the AA levels by ion exchange chromatography (Biotronic LC 3000, Eppendorf, Maintal).

### Statistical analysis

The data analyses were performed using the SAS statistical software package version 7.1 (SAS Inst., Cary, NC, USA). Mean values, as well as the standard deviation (SD), were calculated for all parameters. The study was based on a two-factorial (farrowing system vs. BW) trial. By means of the Shapiro-Wilks test and Kolmogorow-Smirnov test, the parameters were checked for normal distribution. In the case of normally distributed data, the Ryan-Einot-Gabriel-Welsch multiple range test (REGWQ) was used for reproductive parameters (parity, number of total born/ live-born/stillborn piglets, number of piglets after litter equalisation, weaned piglets, piglet losses), IC and daily weight gain to detect significant differences between housing systems. For the availability of normally distributed data, the two-sample t-test was used to compare IC and daily weight gain between light and heavy piglets. To compare the concentrations of the AAs between housing systems and weight classes (light vs. heavy), a nonparametric test (Kruskal-Wallis-test) was used because of their non-normal distribution. This was followed by a pairwise comparison with the Wilcoxon two-sample test. Statistical significance was considered when *p* < 0.05.

## Results

The experiments ran without complications. The specific investigations on serum AA concentrations were carried out on a total of 18 sows and their piglets (a total of 270 live born piglets and 225 weaned piglets; 72 of these piglets were used for blood samples). Of all live born piglets, 45 piglets died during the suckling period and of these piglets 90.0% in FC, 70.8% in LH and 36.7% in GH died in the first three days of life. Of the sampled piglets no piglets of FC died during the first three days of life. In LH two of the sampled piglets died in the first three days and in GH three piglets.

### Reproductive performance

The average reproductive parameters of the sows of the sampled piglets did not differ significantly between the housing groups (Table 1). There were no significant differences in terms of parity, the total number of born piglets, piglets born alive and stillborn piglets. When comparing all sows (*n* = 149) or sows whose piglets were sampled for IC (*n* = 75), the number of weaned piglets was lowest in the LH group and the mortality rate was lowest in the FC group. For sows whose piglets were sampled for AA (*n* = 18), there was no difference in weaned piglets and piglet mortality.

**Table 1:** Reproductive performance of the sows of the sampled piglets, depending on farrowing system (mean ± SD)

	All sows			All Sows with piglets sampled for IC			All sows with piglets sampled for AA		
	FC [*n* = 51]	LH [*n* = 47]	GH [*n* = 51]	FC [*n* = 26]	LH [*n* = 24]	GH [*n* = 25]	FC [*n* = 6]	LH [*n* = 6]	GH [*n* = 6]
Parity number	2.84 ± 2.04	3.43 ± 2.68	2.49 ± 1.86	2.62 ± 1.63	3.13 ± 2.35	2.32 ± 1.25	2.17 ± 0.75	1.67 ± 1.00	2.00 ± 0.89
Total number of born piglets [*n*]	16.3 ± 4.58	15.6 ± 4.44	16.6 ± 4.12	15.9 ± 5.06	15.7 ± 4.72	17.5 ± 4.21	16.5 ± 4.23	17.0 ± 2.10	17.0 ± 3.69
Piglets born alive [*n*]	14.8 ± 4.40	14.2 ± 4.18	15.8 ± 3.90	14.7 ± 4.51	14.5 ± 4.55	16.7 ± 4.07	16.2 ± 3.92	16.2 ± 2.14	16.3 ± 2.94
Stillborn piglets [*n*]	1.49 ± 2.60	1.45 ± 1.65	0.882 ± 0.993	1.23 ± 1.50	1.17 ± 1.58	0.760 ± 0.926	0.33 ± 0.52	0.83 ± 0.98	0.67 ± 0.82
Piglets after litter equalization [*n*]	14.6 ± 2.33	14.2 ± 3.27	15.6 ± 3.21	14.6 ± 2.59	14.5 ± 3.73	16.5 ± 3.61	14.3 ± 2.25	15.3 ± 1.03	15.3 ± 1.86
Weaned piglets [*n*]	12.5^a^ ± 1.86	10.3^b^ ± 2.77	12.0^a^ ± 1.95	12.8^a^ ± 1.91	10.6^b^ ± 2.89	12.6 ± 1.87	12.8 ± 1.94	11.3 ± 2.58	13.3 ± 1.03
Total piglet mortality* [%]	13.2^b^ ± 11.0	24.4^a^ ± 20.1	20.9^a^ ± 14.2	11.1^b^ ± 7.85	23.9^a^ ± 20.2	20.9^a^ ± 15.1	10.2 ± 6.40	26.4 ± 15.5	12.1 ± 11.5

*FC* farrowing crate, *LH* Loose housing, *GH* Group housing

^a,b^ Values within a row with different superscripts differ significantly at *p* < 0.05

* Piglet mortality detected the period from birth to weaning. Piglets moved away due to the litter equalization are not recorded as losses. All piglets of a litter that died during the total suckling period (apart from the piglets that were transferred), describe the piglet mortality

The significantly lowest average daily litter gain was determined in GH (in kg; FC (Ø 13.0 piglets/sow): 3.03^ab^, LH (Ø 11.4 piglets/sow): 3.13^a^, GH (Ø 13.5 piglets/sow): 2.89^b^).

### Immunocrit

The determined total IC was significantly lower in GH (from piglets of all sows sampled for IC: – 15.8% compared to FC, − 14.7% compared to LH; from piglets of all sows sampled for AA: - 20.9% compared to FC; − 23.5% compared to LH; Table 2). The IC of the light piglets was significantly different between LH and GH. The light piglets in GH showed the lowest IC (from piglets of all sows sampled for IC: – 18.2% compared to FC, − 16.6% compared to LH; from piglets of all sows sampled for AA: - 17.3% compared to FC, − 28.6% compared to LH). The significantly lowest IC of the heavy piglets could be seen in GH (from piglets of all sows sampled for IC: – 11.5% compared to FC, − 9.88% compared to LH; from piglets of all sows sampled for AA: - 20.0% compared to FC, − 19.0% compared to LH).

**Table 2:** BW 24 h p.n. and IC of piglets within 48 h p.n. depending on farrowing system (mean ± SD)

	*n*	BW [kg]	IC	*n*	BW [kg]	IC	*n*	BW [kg]	IC
All sows with piglets sampled for IC									
	FC (26 sows)			LH (24 sows)			GH (25 sows)		
Light	45	1.11^B^ ± 0.087	0.148^Ba^ ± 0.034	35	1.16^B^ ± 0.106	0.145^Ba^ ± 0.037	50	1.15^B^ ± 0.111	0.121^Bb^ ± 0.038
Heavy	60	1.80^A^ ± 0.258	0.165^Aa^ ± 0.027	61	1.82^A^ ± 0.275	0.162^Aa^ ± 0.034	48	1.87^A^ ± 0.270	0.146^Ab^ ± 0.038
Total	105	1.50 ± 0.398	0.158^a^ ± 0.032	96	1.58 ± 0.394	0.156^a^ ± 0.030	98	1.51 ± 0.417	0.133^b^ ± 0.040
All sows with piglets sampled for AA									
	FC (6 sows)			LH (6 sows)			GH (6 sows)		
Light	8	1.10^B^ ± 0.036	0.133^ab^ ± 0.030	10	1.16^B^ ± 0.104	0.154^a^ ± 0.033	12	1.16^B^ ± 0.112	0.110^b^ ± 0.032
Heavy	16	1.78^A^ ± 0.235	0.155^a^ ± 0.031	14	1.77^A^ ± 0.239	0.153^a^ ± 0.029	12	1.96^A^ ± 0.319	0.124^b^ ± 0.038
Total	24	1.56 ± 0.382	0.148^a^ ± 0.032	24	1.52 ± 0.360	0.153^a^ ± 0.030	24	1.56 ± 0.473	0.117^b^ ± 0.029

FC farrowing crate, LH loose housing, GH group housing, BW body weight [kg], IC immunocrit; light < 1.4 kg, heavy ≥1.4 kg

^a.b^ Values within a row with different superscripts differ significantly at *p* < 0.05; ^A.B^Values within a column with different superscripts differ significantly at *p* < 0.05

No significant differences between light and heavy piglets regarding the IC could be observed independent of the housing system for all piglets of sows sampled for AA in serum (Table 3).

**Table 3:** BW 24 h p.n and IC of piglets from sows with piglets sampled for AA within 48 h p.n. depending on weight class (mean ± SD)

		*N*	BW [kg]	IC
within 48 h p.n.	Light	30	1.14^B^ ± 0.10	0.131 ± 0.036
	Heavy	42	1.83^A^ ± 0.27	0.146 ± 0.032

*BW* Body weight [kg], *IC* Immunocrit; light < 1.4 kg. heavy ≥1.4 kg

^A.B^ Values within a column with different superscripts differ significantly at *p* < 0.05

### Amino acid concentrations post natum and at weaning

The piglets in GH, independent of BW, showed a significantly lower concentration of leucine (− 32.0% compared to FC; − 24.7% compared to LH), proline (− 22.6% compared to FC; − 32.6% compared to LH) and threonine (− 28.2% compared to FC; − 30.4% compared to LH; Table 4). The levels of arginine differed significantly between LH and GH (− 30.8% compared to LH). Significant differences could be seen between FC and GH regarding the concentrations of lysine (− 32.8% compared to FC) and valine (− 24.1% compared to FC).

**Table 4:** Serum AAs and NH_3_ [mg/dL] in piglets within 48 h p.n. depending on farrowing system (mean ± SD)

	FC [*n* = 24]	LH [*n* = 24]	GH [*n* = 24]
BW [kg]	1.56 ± 0.38	1.52 ± 0.360	1.56 ± 0.473
Serum AAs [mg/dL]			
Arginine	2.65^ab^ ± 1.44	2.53^a^ ± 1.09	1.75^b^ ± 0.44
Leucine	3.09^a^ ± 1.06	2.79^a^ ± 1.05	2.10^b^ ± 0.60
Lysine	5.19^a^ ± 2.62	4.04^ab^ ± 2.07	3.49^b^ ± 1.21
Methionine	0.96 ± 0.76	0.85 ± 0.82	0.67 ± 0 32
Proline	10.2^a^ ± 3.86	11.7^a^ ± 3.67	7.89^b^ ± 3.07
Threonine	2.77^a^ ± 1.17	2.86^a^ ± 0.86	1.99^b^ ± 0.92
Tryptophan^*^	0.64 ± 0.18	0.64 ± 0.32	0.57 ± 0.18
Valine	5.10^a^ ± 1.85	4.87^ab^ ± 2.01	3.87^b^ ± 1.07
NH_3_	6.61 ± 1.35	7.16 ± 1.30	7.35 ± 1.39

FC farrowing crate, LH loose housing, GH group housing, BW body weight [kg]

^a,b^ Values within a row with different superscripts differ significantly at *p* < 0.05

^*^ FC: *n* = 8; LH: *n* = 8; GH: *n* = 7 (only measurable with these piglets)

No significant differences could be seen between light and heavy piglets with regard to AA levels within a housing system (Table 5).

**Table 5:** Serum AAs and NH_3_ [mg/dL] in piglets within 48 h p.n. depending on farrowing system and weight class (mean ± SD)

		FC	LH	GH
Light	n	8	10	12
	BW [kg]	1.10^B^ ± 0.04	1.16^B^ ± 0.10	1.16^B^ ± 0.11
	Serum AAs [mg/dL]			
	Arginine	2.10^ab^ ± 0.90	2.65^a^ ± 1.27	1.57^b^ ± 0.39
	Leucine	2.85^a^ ± 0.76	2.72^ab^ ± 0.89	2.01^b^ ± 0.70
	Lysine	4.92 ± 2.47	4.01 ± 1.26	3.43 ± 1.34
	Methionine	0.80 ± 0.22	0.72 ± 0.67	0.59 ± 0.30
	Proline	10.5^ab^ ± 3.67	12.1^a^ ± 2.92	8.42^b^ ± 3.45
	Threonine	2.30 ± 0.99	2.77 ± 1.0	1.83 ± 1.09
	Tryptophan^*^	0.60 ± 0.09	0.59 ± 0.24	0.67 ± 0.20
	Valine	4.35 ± 0.98	4.66 ± 1.96	3.74 ± 1.40
	NH_3_	6.68 ± 0.99	6.91 ± 1.42	7.45 ± 1.52
Heavy	n	16	14	12
	BW [kg]	1.78^A^ ± 0.24	1.77^A^ ± 0.24	1.96^A^ ± 0.32
	Serum AAs [mg/dL]			
	Arginine	2.93 ± 1.60	2.45 ± 0.98	1.92 ± 0.42
	Leucine	3.21^a^ ± 1.19	2.84^ab^ ± 1.19	2.19^b^ ± 0.50
	Lysine	5.32 ± 2.76	4.07 ± 2.55	3.55 ± 1.12
	Methionine	1.03 ± 0.92	0.94 ± 0.93	0.80 ± 0.31
	Proline	10.0^ab^ ± 4.06	11.5^a^ ± 4.22	7.37^b^ ± 2.69
	Threonine	3.00^ab^ ± 1.20	2.92^a^ ± 0.77	2.15^b^ ± 0.72
	Tryptophan^**^	0.66 ± 0.23	0.68 ± 0.39	0.49 ± 0.14
	Valine	5.47 ± 2.08	5.03 ± 2.10	4.01 ± 0.61
	NH_3_	6.58 ± 1.53	7.33 ± 1.24	7.26 ± 1.31

*FC* Farrowing crate, *LH* Loose housing, *GH* Group housing, *BW* Body weight [kg]; light < 1.4 kg. heavy ≥1.4 kg

^a,b^Values within a row with different superscripts differ significantly at *p* < 0.05; ^A,B^Values within a column with different superscripts differ significantly at *p* < 0.05

^*^FC: *n* = 3; LH: *n* = 3; GH: *n* = 3; ^**^ FC: *n* = 5; LH: *n* = 5; GH: *n* = 4 (only measurable with these piglets)

#### Light piglets

The levels of AAs of the light piglets differed significantly between LH und GH with respect to the concentrations of arginine (+ 40.8% compared to GH) and proline (+ 30.4% compared to GH). The light piglets in FC had a significantly higher leucine concentration compared to GH (+ 29.5%).

#### Heavy piglets

There were significant differences between the FC and GH regarding the leucine level (+ 31.8% compared to GH). Levels of proline and threonine differed significantly between LH und GH (compared to GH: proline + 35.9%; threonine: + 26.4%).

Significantly highest BW of the piglets on the day of weaning (day 26), independent of weight class could be seen in LH (Table 6). Levels of leucine (− 17.6% compared to FC; − 22.4% compared to GH), proline (− 13.4% compared to FC; − 18.0% compared to GH) and valine (− 21.1% compared to FC; − 24.0% compared to GH) were significantly lower in LH. Significant differences could be observed between FC and GH regarding the average levels of arginine (compared to FC: arginine - 20.1%; threonine - 30.6%). The level of lysine differed significantly between LH and GH (− 23.0% compared to GH).

**Table 6:** Serum AAs and NH_3_ [mg/dL] in piglets on weaning depending on farrowing system (mean ± SD)

	FC [*n* = 24]	LH [*n* = 22]	GH [*n* = 21]
BW [kg]	8.20^ab^ ± 2.22	9.05^a^ ± 2.05	7.33^b^ ± 2.49
Serum AAs [mg/dL]			
Arginine	3.68^a^ ± 0.71	3.40^ab^ ± 0.73	2.94^b^ ± 0.77
Leucine	2.73^a^ ± 0.62	2.25^b^ ± 0.45	2.90^a^ ± 0.53
Lysine	3.42^ab^ ± 0.99	2.85^b^ ± 0.69	3.70^a^ ± 1.31
Methionine	1.11 ± 0.39	1.09 ± 0.28	1.17 ± 0.62
Proline	7.33^a^ ± 1.55	6.35^b^ ± 1.23	7.74^a^ ± 1.88
Threonine	3.59^a^ ± 1.38	3.02^ab^ ± 1.08	2.49^b^ ± 0.94
Tryptophan^*^	0.44 ± 0.59	0.56 ± 0.49	0.48 ± 0.51
Valine	3.17^a^ ± 0.56	2.50^b^ ± 0.49	3.29^a^ ± 1.07
NH_3_	7.62 ± 1.23	7.22 ± 0.87	7.20 ± 1.89

FC farrowing crate, LH loose housing, GH group housing, BW body weight [kg]

^a,b^ Values within a row with different superscripts differ significantly at *p* < 0.05

^*^ FC: *n* = 6; LH: *n* = 7; GH: *n* = 5 (only measurable with these piglets)

On the day of weaning, between light and heavy piglets in FC, significant differences could be observed regarding the mean levels of proline (+ 16.9% compared to heavy piglets; Table 7). Heavy piglets in LH showed a significantly higher concentration of valine compared to their lighter littermates (+ 18.3%).

**Table 7:** Serum AAs and NH_3_ [mg/dL] in piglets on weaning depending on farrowing system and weight class (mean ± SD)

		FC	LH	GH
Light	n	8	8	10
	BW [kg]	6.26^Bab^ ± 1.14	7.74^Ba^ ± 1.71	5.31^Bb^ ± 1.62
	Serum AAs [mg/dL]			
	Arginine	3.45 ± 0.81	3.41 ± 0.74	2.75 ± 0.96
	Leucine	2.70^a^ ± 0.58	2.07^b^ ± 0.37	2.75^a^ ± 0.53
	Lysine	3.30^ab^ ± 0.89	2.72^b^ ± 0.67	3.48^a^ ± 0.78
	Methionine	1.24 ± 0.41	1.05 ± 0.37	1.28 ± 0.72
	Proline	8.27^Aa^ ± 1.14	6.29^b^ ± 1.38	7.29^ab^ ± 2.36
	Threonine	3.99^a^ ± 1.51	2.92^ab^ ± 1.44	2.63^b^ ± 1.01
	Tryptophan^*^	0.78 ± 0.34	0.93 ± 0.34	0.95 ± 0.26
	Valine	3.14^a^ ± 0.45	2.19^Bb^ ± 0.29	3.50^a^ ± 0.89
	NH_3_	8.40 ± 1.35	7.15 ± 1.04	8.00 ± 1.79
Heavy	n	16	14	11
	BW [kg]	9.18^A^ ± 1.97	9.81^A^ ± 1.88	8.97^A^ ± 1.75
	Serum AAs [mg/dL]			
	Arginine	3.80^a^ ± 0.65	3.40^ab^ ± 0.75	3.11^b^ ± 0.54
	Leucine	2.75^a^ ± 0.66	2.35^b^ ± 0.48	3.03^a^ ± 0.52
	Lysine	3.49^ab^ ± 1.06	2.92^b^ ± 0.71	4.13^a^ ± 1.19
	Methionine	1.11 ± 0.25	1.12 ± 0.22	1.15 ± 0.40
	Proline	6.87^Ba^ ± 1.54	6.39^a^ ± 1.19	8.14^b^ ± 1.30
	Threonine	3.39^a^ ± 1.31	3.08^ab^ ± 0.86	2.36^b^ ± 0.89
	Tryptophan^**^	1.03 ± 0.54	0.84 ± 0.25	0.89 ± 0.33
	Valine	3.19^a^ ± 0.62	2.68^Ab^ ± 0.50	3.39^a^ ± 0.68
	NH_3_	7.24 ± 0.99	7.25 ± 0.80	6.47 ± 1.74

*FC* Farrowing crate, *LH* Loose housing, *GH* Group housing, *BW* Body weight [kg], light (birth weight 24 h p.n.) < 1.4 kg, heavy (birth weight 24 h p.n.) ≥ 1.4 kg

^a,b^ Values within a row with different superscripts differ significantly at p < 0.05; ^A,B^ Values within a column with different superscripts differ significantly at p < 0.05

^*^ FC: *n* = 3; LH: *n* = 5; GH: *n* = 4; ^**^ FC: *n* = 8; LH: *n* = 9; FC: *n* = 7 (only measurable with these piglets)

#### Light piglets

The light piglets on the day of weaning in GH showed the significantly lowest BW compared to LH (in kg; − 2.43). Concentrations of leucine (− 23.3% compared to FC; − 24.7% compared to GH) and valine (− 30.3% compared to FC; − 37.4% compared to GH) were the lowest in LH. There was a significant difference with regard to lysine level between LH and GH (− 21.8% compared to GH). The proline concentration differed significantly between FC and LH (− 23.9% compared to FC). Light piglets in GH had the significantly lowest threonine level compared to FC (− 34.1%).

#### Heavy piglets

Levels of leucine (− 14.5% compared to FC; − 22.4% compared to GH) and valine (− 16.0% compared to FC; − 20.9% compared to GH) were significantly lowest in LH. Piglets in GH showed the significantly highest proline level (in mg/dL; + 1.27 compared to FC; + 1.75 compared to LH). The concentrations of arginine and threonine differed significantly between FC and GH (compared to FC: arginine - 18.2%; threonine - 30.4%). There was a significant difference regarding the lysine level between LH and GH (− 29.3% compared to GH).

Table 8 shows the AA levels in piglets’ serum within 48 h p.n. and on the day of weaning divided into light and heavy piglets independent of the farrowing system. At both times, no significant differences in AA concentrations could be observed between light and heavy piglets.

**Table 8:** Serum AAs and NH_3_ [mg/dL] in piglets within 48 h p.n. and on weaning depending on weight class (mean ± SD)

	within 48 h p.n.		day of weaning (day 26)	
	Light [*n* = 30]	Heavy [*n* = 42]	Light [*n* = 26]	Heavy [*n* = 41]
BW [kg]	1.14^b^ ± 0.38	1.83^a^ ± 0.36	6.39^b^ ± 0.47	9.34^a^ ± 0.47
Serum AAs [mg/dL]				
Arginine	2.07 ± 0.99	2.48 ± 1.21	3.17 ± 0.88	3.48 ± 0.70
Leucine	2.47 ± 0.85	2.79 ± 1.10	2.53 ± 0.57	2.69 ± 0.61
Lysine	4.02 ± 1.73	4.40 ± 2.40	3.19 ± 0.82	3.47 ± 1.08
Methionine	0.69 ± 0.44	0.94 ± 0.78	1.20 ± 0.53	1.12 ± 0.28
Proline	10.2 ± 3.59	9.75 ± 4.04	7.28 ± 1.88	7.05 ± 1.50
Threonine	2.27 ± 1.08	2.73 ± 1.00	3.14 ± 1.39	3.01 ± 1.12
Tryptophan^*^	0.62 ± 0.17	0.62 ± 0.27	0.90 ± 0.29	0.92 ± 0.39
Valine	4.21 ± 1.53	4.91 ± 1.86	2.98 ± 0.83	3.07 ± 0.66
NH_3_	7.07 ± 1.36	7.02 ± 1.39	7.86 ± 1.49	7.04 ± 1.20

BW body weight [kg]; light (birth weight 24 h p.n.) < 1.4 kg, heavy (birth weight 24 h p.n.) ≥ 1.4 kg

^a,b^Values within a row with different superscripts differ significantly at p < 0.05

^*^within 48 h p.n. Light: *n* = 9; Heavy: *n* = 14. day of weaning Light: *n* = 12. Heavy: *n* = 24 (only measurable with these piglets)

### Growth performance of piglets

**Fig. 4:**
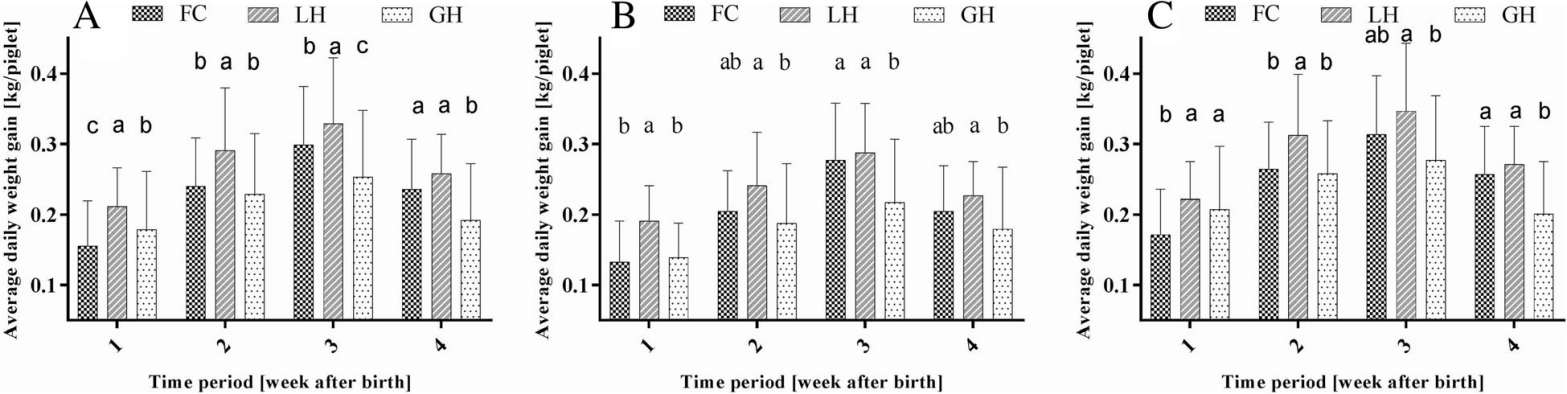
Daily weight gain depending on farrowing system (FC, LH, GH). **a** = of all piglets of the sampled sows; **b** = of the light piglets (< 1.4 kg); C = of the heavy piglets (≥ 1.4 kg); (FC farrowing crate, LH loose housing, GH group housing)

Figure 4 shows the development of the average daily weight gain of all piglets (A), light piglets (B) and heavy piglets (C) of the sampled eighteen litters during the suckling period depending on the farrowing system. In (A), the highest average daily weight gain could be observed at week level in LH. However, in the last week of the suckling period, no significant differences could be seen any longer between FC and GH (in g; FC: 236^a^, LH: 258^a^; GH: 192^b^). Apart from the first week of lactation, piglets in group housing showed the lowest steady daily weight gain.

Average daily weight gain of light piglets in general differed significantly between LH and GH (Fig. 4; (B)). Except for the first week, the growth performance did not differ between FC and LH. Numerically, heavy piglets in LH showed the highest average daily weight gain during the four weeks (Fig. 4; (C)). The individual BWs of the sampled piglets on the five weighing dates are displayed in an additional file (see Additional file 1).

## Discussion

### Reproductive performance

The investigations were conducted without incidents. The number of piglets born alive to the sows were consistent with the results of agricultural farms in Northern Germany in 2016/17 [35]. Regarding the number of weaned piglets per litter in FC (12.8) and GH (13.3) in sows of piglets analysed for AA level in serum, similar results as in practice could also be achieved [35]. The number of weaned piglets in LH recorded in the present study (11.3 piglets/litter) was considerably less than that reported in pig farming (13.1 piglets/litter; [35]), which is probably due to the unacceptably numerically higher piglet losses in this housing system (26.4%). Piglet losses (calculated from birth to weaning) in FC (10.2%) and GH (12.1%) were comparable with those observed in 2016/17 (15.1%) in Northern Germany [35]. Nevertheless, the piglet losses of all 149 sows were significantly lower in FC compared to the other two housing systems (FC: 13.2^b^, LH: 24.4^a^, GH: 20.9^a^, respectively). According to Hales et al. [33] and Chidgey et al. [36], alternative free farrowing systems are often associated with higher piglet mortality. Similarly, high piglet losses of about 23.1% in a free farrowing pen could also be observed in another report [37]. Pre-weaning mortality rate of about 22.6% was observed in a free farrowing system with a communal area [38]. Nevertheless, there are studies that did not determine any higher piglet losses (1.40 piglets (loose) vs. 1.42 piglets (crate)) in alternative farrowing systems [39]. Overall, the comparison of piglet losses is fundamentally difficult due to the diversity of different alternative farrowing systems. Individual differences in maternal behaviour, which is an important factor for piglet survival [40], may also have led to these differences between systems. In conclusion, we were not able to achieve acceptable production results in the tested new systems (LH, GH). Therefore, the alternative systems still need to be improved and further parameters may indicate why there are differences between these housing systems.

### Immunocrit

Pre-weaning mortality and poor performance of piglets also often result from low colostrum intake [10, 14, 41]. Immediately after birth, newborn piglets have limited energy stores and are therefore dependent on the intake of colostrum [5, 42]. Inadequate colostrum intake is described as one of the most important factors influencing the survival chances of piglets [2]. The IC values in the first 48 h p.p. of the lighter piglets (< 1.4 kg) were lower than in their heavier (≥ 1.4 kg) littermates. In studies by Quesnel et al. [41] and Devillers et al. [43], it could be shown that lighter piglets absorb less colostrum. The birth weight and body reserves of the individual piglets decrease as the litter sizes become larger and larger [2, 9]. These piglets often show poor performance resulting from a low colostrum intake [44–46]. Looking at the results from litters that have been comprehensively investigated (including AA), the total IC within 48 h p.n. was significantly lower in GH by - 20.9% compared to FC and − 23.5% compared to LH. The light piglets in GH had an average IC which was significantly lower compared to LH (− 28.6%) and numerically lower compared to FC (− 17.3%). The IC values of the heavy piglets were significantly higher in FC (+ 20.0%) and LH (+ 19.0%) compared to GH. In general, there are clear indications that neonates in GH showed the lowest Ig levels. Morton et al. [47] examined the influence of split nursing on the IC. The piglets in the control group (all pigs suckled ad libitum; BW <  1.45 kg) showed an average IC of 0.150 [47]. In comparison to this, heavy piglets of the second treatment (weight based split suckling: the heaviest six pigs were removed for 1.5 h) had an average IC of 0.147 [47]. The difference was therefore only 2.0% between treatments [47]. Thus, the type of housing system in the present study seems to have a much greater influence on the Ig levels. Potential factors that could contribute to piglets ingesting lower levels of colostrum are the parity as well as the number of piglets born alive to the sow [48]. In the present study, no significant differences could be seen concerning these parameters between housing systems concerning reproductive performance (Table 1). Due to a selection of certain animals, factors such as parity and litter size could be standardised, which in turn allowed a better evaluation of the different housing systems themselves. The reason for the lower immunocrit levels remains unexplained. The group housing in the peripartal period of the sows probably led to increased ranking fights, which can result in increased stress [49]. Stress can have a negative impact on colostrum formation [50]. From literature, it is known that increased stress shortly before birth leads to significantly lower IgG concentrations in the colostrum and in piglets´ blood [51]. In an early study on suckling behaviour of piglets, Weary et al. [52] observed a decrease in suckling frequency of sows in group housing during lactation. In the present study, differences in the suckling behaviour between systems could be one explanation for the significantly lower Ig levels of piglets in GH.

An exact explanation for the low IC of piglets in specific housing systems in this study cannot be found. Even if the feed intake before birth is considered, there is hardly no reason for differences. The feed intake could not be accurately recorded for all sows due to a loss of data owing to a hardware problem. The actual feed intake across all recorded sows was similar (day − 5 – 0, in kg/DM; FC: 16.7 (48 sows), LH: 16.4 (37 sows), GH: 17.2 (21 sows)). For the 18 sows in the study, the differences were a little bit higher (day − 5 – 0, in kg/DM; FC: 18.7, LH: 17.5, GH: 26.5). Therefore, basically the feed intake was higher in the group GH before birth. Therefore, an influence on the onset of lactation cannot be ruled out. Further investigations would be necessary here. However, it is difficult to keep a group with very small amounts of feed in the last days before birth calm, because otherwise the animals tend to be restless. In general, it’s amount was nearly independent of the feeding system even if ad libitum intake was possible (GH). Yun et al. [53] also pointed out other important relationships that have to be considered when evaluating a system. In their study, piglet growth in litters from sows confined in crates before and during farrowing was lower than of sows kept in loose-housed pens during the same period. They stated that specific adaptations in the management (for example provision of nesting materials) could increase serum IgG and IgM concentrations in piglets. From our study it is not clear, whether results would have been different if sows had been acclimatised to the specific systems from the first farrowing onwards. Further investigations should clarify if pen layout itself, stress or management might be a reason for lower performance of sows in GH.

### Amino acid concentrations post natum and at weaning and growth performance

Piglets in GH post natum showed the lowest AA concentrations in blood samples. Arginine (in mg/dL; FC: 2.65^ab^, LH: 2.53^a^, GH: 1.75^b^) and leucine (in mg/dL; FC: 3.09^a^, LH: 2.79^a^, GH: 2.10^b^) for example were lower in GH. A previous study shows that a dietary arginine supplementation (0.2% L-arginine on the basis of milk replacer powder) of seven-day-old piglets increased arginine plasma levels by 30% compared to the control pigs [28]. Between days 7–21, a significant difference could be seen regarding the daily weight gain (in g/day; 180 vs. 230) between the control and arginine group. Additionally, Sun et al. [25] reported that L-leucine supplementation (between seven and 21 days of age) improves the development of the intestinal tract, which results in higher absorption of dietary nutrients and higher weight gains [25]. The average daily weight gain between days 7–21 was higher (*p* = 0.004) in the leucine-group (in g/d; 238 vs. 265) than in the control group. Plasma levels of leucine were also higher (*p* < 0.01) in the leucine group (in μM; 311 vs. 192). These results indicate that higher serum AA levels could have a great influence on piglets’ performance. Furthermore, piglets with higher BW showed higher serum AA concentrations in the present study. This is in agreement with the findings of Decaluwé et al. [54], where colostrum intake per kg birth weight was positively associated with some free AA (valine *p* = 0.03; leucine *p* = 0.02) in the serum of piglets. Basically, the concentrations of amino acids in the blood depend on the absolute intake of nutrients and the utilization in the first pass by the intestine and the liver [55]. In general, one third of the dietary essential AA intake is metabolised in the first pass by the intestinal mucosa [55]. For same AA, the net portal balance exceeds the intake (arginine: 137%; [55]). It has to be taken into account, that the quantities of dietary amino acids utilized in the first pass by the intestine are closely related to the mucosal mass of the piglets [55]. Therefore, it cannot be clearly concluded, whether low concentrations in the blood are due to high first pass utilization, low intake or both. Therefore, the ammonia concentration in the blood is an additional parameter to be mentioned. The net portal outflow of ammonia is responsible for about 18% of the amino acid nitrogen intake [55]. In this study, there was no difference in blood NH_3_-concentrations. Therefore, it is not clear, if the lower AA concentrations in GH are responsible for a somewhat poor intestinal development and as a consequence, piglets were unable to realise their growth potential and instead low weight gains could be observed. Colostrum intake and therefore a high level of passive immunity of piglets has a long-term effect on health which also plays an important role in weight gain and performance [43]. However, before weaning, piglets in GH showed only the numerically lowest AA levels of arginine and threonine. The significantly lowest concentrations of leucine, lysine, proline and valine could be seen in LH. In principle, the results of AA levels from the day of weaning show that the housing system itself does not have a lasting adverse effect on the level of free AAs in the blood and might not impair piglets’ health in the long run. Only lower weight gains could be observed in these piglets at weaning. Nevertheless, if in future group housing of lactating sows is considered, further investigations have first to be carried out to ensure that newborn piglets in such housing systems are adequately supplied with colostrum immediately after birth. At the same time, the long-term growth behaviour of piglets could be an interesting topic.

## Conclusion

In conclusion, results reported here indicate that different farrowing systems could influence the Ig levels and therefore might have an influence on the performance of piglets. The tested group housing had a negative effect on IC and on AA levels post natum in serum. Nevertheless, no negative influence on AAs of the piglets in GH could be observed on the day of weaning. From our study it is not clear, whether results would have been different if sows had been acclimatised to the specific systems from the first farrowing onwards. In addition, no management measures specific to the respective systems were established. These could possibly lead to significant improvements in the LH and GH systems. Therefore, each system needs its specific strategy especially for fostering colostrum supply in low birth weight piglets.

## Additional file

Additional file 1: ‘BW [kg] of the sampled piglets at five different weighing times during suckling period’. Additional file shows individual BW of the sampled piglets (light/heavy/total) at five different weighing points. (PDF 281 kb)

## Data Availability

The datasets used and/or analysed during the current study are available from the corresponding author on reasonable request.

## Abbreviations

AA: Amino acid
BW: Body weight
FC: Farrowing crate
GH: Group housing
IC: Immunocrit
Ig: Immunoglobulin
LH: Loose housing
p.n.: *post natum*
p.p.: *post partum*
