# Supplementary material for: Immunocrit, serum amino acid concentrations and growth performance in light and heavy piglets depending on sow’s farrowing system
Source: Porcine Health Manag. 2019 Jun 14;5:14. doi: 10.1186/s40813-019-0121-1 (PMC6567442; doi:10.1186/s40813-019-0121-1)
Supplement: Supplementary file 1 — ‘BW [kg] of the sampled piglets at five different weighing times during suckling period’. Additional file shows individual BW of the sampled piglets (light/heavy/total) at five different weighing points. (PDF 281 kb) [file 40813_2019_121_MOESM1_ESM.pdf]

Additional file 1. BW [kg] of the sampled piglets on five different weighing times during suckling period

		FC		LH		GH	
		n	kg	*n*	kg	*n*	kg
**24h p.n.**	**light**	8	1.10 ± 0.04	10	1.16 ± 0.10	12	1.16 ± 0.11
	**heavy**	16	1.78 ± 0.23	14	1.77 ± 0.24	12	1.96 ± 0.32
	**Total Ø**	24	1.56 ± 0.38	24	1.52 ± 0.36	24	1.56 ± 0.47
**day 7 p.n.**	**light**	8	2.43 ± 0.61	8	2.29 ± 0.37	10	2.04 ± 0.55
	**heavy**	16	3.68 ± 0.97	14	3.10 ± 0.49	11	3.73 ± 1.91
	**Total Ø**	24	3.26 ± 1.04	22	2.81 ± 0.59	21	2.93 ± 1.65
**day 14 p.n.**	**light**	8	2.90^{b} ± 0.42	8	4.28^{a} ± 0.81	9	3.02^{b} ± 0.65
	**heavy**	16	4.57^{b} ± 0.78	14	5.50^{a} ± 0.83	11	5.23^{a} ± 0.62
	**Total Ø**	24	4.01^{b} ± 1.04	22	5.05^{a} ± 1.00	20	4.24^{b} ± 1.29
**day 21 p.n.**	**light**	8	4.71^{b} ± 0.73	8	6.44^{a} ± 1.31	9	4.18^{b} ± 1.18
	**heavy**	16	6.86^{b} ± 1.32	14	8.11^{a} ± 1.43	11	7.47^{ab} ± 1.04
	**Total Ø**	24	6.14^{b} ± 1.54	22	7.50^{a} ± 1.59	20	5.99^{b} ± 2.00
**weaning**	**light**	8	6.26^{ab} ± 1.14	8	7.74^{a} ± 1.71	9	5.31^{b} ± 1.62
	**heavy**	16	9.18 ± 1.97	14	9.81 ± 1.88	11	8.97 ± 1.75
	**Total Ø**	24	8.20^{ab} ± 2.22	22	9.05^{a} ± 2.05	20	7.33^{b} ± 2.49

^{a,b}Values within a row with different superscripts differ significantly at $P < 0.05$
